# The Serotonin-Mediated Anti-Allodynic Effect of Yokukansan on Paclitaxel-Induced Neuropathic Pain

**DOI:** 10.3390/medicina60030359

**Published:** 2024-02-21

**Authors:** Hiroshi Yokomi, Takahiro Kato, Soshi Narasaki, Satoshi Kamiya, Shima Taguchi, Yosuke T. Horikawa, Yasuo M. Tsutsumi

**Affiliations:** Department of Anesthesiology and Critical Care, Hiroshima University Hospital, Hiroshima 734-8551, Japan

**Keywords:** Yokukansan, Paclitaxel, 5-HT receptor, neuropathic pain

## Abstract

Refractory peripheral neuropathy can occur as a side effect in 60–70% of patients receiving Paclitaxel (PTX). Yokukansan (YKS) is a Japanese herbal medicine reported to have analgesic properties for entrapment nerve injuries. Therefore, we investigated the anti-allodynic effect of Yokukansan on Paclitaxel-induced neuropathic pain. All experiments used 6-week-old male Sprague Dawley rats. Mechanical allodynia was evaluated using a dynamic plantar aesthesiometer. A mobile touch-stimulator unit applied progressively increasing force to the mid-plantar region of the hind paw in a vertical direction until the animal withdrew its paw. This was carried out before the Paclitaxel administration and during the first, second, third, and fourth weeks. Using a rat model of PTX-induced neuropathic pain (PTX rat), we injected PTX (intraperitoneally, 2 mg/kg) five times every 2 days. Using the dynamic plantar test, we evaluated the anti-allodynic effect of YKS (orally administered, 1 g/kg). YKS administration on a daily basis significantly enhanced the withdrawal threshold in PTX rats and reduced the expression level of activated microglia immunostaining with Iba1, a specific marker for microglia. The intrathecal administration of WAY-100635 (5-hydroxytryptamine [5-HT]_1A_ receptor antagonist) and Ketanserin (5-HT_2A/2C_ receptor antagonist) inhibited the protective effects of YKS. YKS exhibited an anti-allodynic effect in a rodent model of PTX-induced neuropathic pain by reducing the sensitivity to pain stimuli. These results suggest that Yokukansan may activate 5-HT receptors in the spinal cord, mediating Paclitaxel-induced neuropathic pain.

## 1. Introduction

Chemotherapy with anticancer medications is essential to current cancer treatment protocols, but peripheral neuropathy, including pain and numbness, is a major problem for patients. Chemotherapy-induced peripheral neuropathy can occur with a relatively high frequency and can significantly impair a patient’s quality of life [1]. Furthermore, peripheral neuropathy may result in suboptimal treatment doses, as well as the discontinuation of chemotherapy. The peripheral neuropathy caused by chemotherapy often persists even after administration and is difficult to improve with conventional treatments. Therefore, there is a strong demand for new preventive methods and therapeutic methods.

Paclitaxel (PTX) is one of the most widely used anticancer drugs and is approved for use in solid tumor cancers, such as non-small cell lung cancer and ovarian cancer [2,3]. Refractory peripheral neuropathy can occur as a side effect in 60–70% of patients receiving PTX [4]. It has a high affinity for tubulin and promotes microtubule polymerization in peripheral nerve axons to form abnormal microtubule bundles. PTX-induced neuropathy is thought to occur because PTX interferes with normal axonal transport [5].

Yokukansan (YKS) is a traditional Japanese herbal medicine (Kampo) with centuries of history. It consists of a blend of seven herbs and is known for its calming and sedative effects, making it particularly useful in managing various neuropsychiatric symptoms [6]. These herbs are combined in specific proportions to create Yokukansan, used in traditional Japanese medicine for various purposes, including treating behavioral and psychological symptoms of dementia [7]. YKS is primarily known for its calming and sedative effects on the central nervous system. It is used to alleviate symptoms associated with neuropsychiatric conditions, such as anxiety, insomnia, irritability, agitation, and behavioral and psychological symptoms of dementia. Studies have suggested that YKS may exert its effects through various mechanisms, including the modulation of neurotransmitter systems and neuroinflammation [8]. YKS has been reported to show an analgesic effect on the entrapment nerve injury pain model [7]. A mechanism of YKS’s analgesic effect is its activating the descending pain inhibitory system in the spinal cord through the partial agonist action of serotonin [9].

However, the effect of YKS on PTX-induced neuropathic pain is unclear. Therefore, we investigated the anti-allodynic effect of YKS on PTX-induced neuropathic pain. We also examined the involvement of serotonin in the spinal cord, which is one of the analgesic sites of YKS, by administering serotonin receptor antagonists into the medullary cavity.

## 2. Materials and Methods

### 2.1. Animals

Male Sprague Dawley rats, aged 6 weeks and weighing 250–300 g, were obtained from Charles River Laboratories Japan, Inc. (Yokohama-shi, Japan). All animals were housed under clean conditions with a controlled temperature (23 ± 2 degrees) and a 12 h light–dark cycle (lights were on from 08:00 to 20:00 h) and were provided food and water ad libitum. All experiments were performed in the same time window to avoid changes in physiological rhythms. The total number of animals used was 90. All animal experimental procedures (approval number: A21-10) were approved by the Committee of Animal Experimentation, Hiroshima University. *The Guide for the Care and Use of Laboratory Animals* was utilized to properly handle and care for the animals.

### 2.2. Drugs

The dosing formulations were freshly prepared on the day of use. The PTX and YKS were purchased from Nippon Kayaku Co. (Tokyo, Japan) and Tsumura & Co. (Tokyo, Japan). YKS consists of seven herbs: Atractylodes lancea rhizome, Poria sclerotium, Cnidium rhizome, Uncaria hook, Angelica root, Bupleurum root, and Glycyrrhiza (Table 1). 4-Chloro-DL-phenylalanine methyl ester hydrochloride (Sigma, St. Louis, MO, USA) was used as a serotonin synthesis inhibitor. Each of the 5-hydroxytryptamine (5-HT) receptor antagonists, WAY-100635 (5-HT_1A_ receptor antagonist; Abcam, Tokyo, Japan), Ketanserin (5-HT_2A/2C_ receptor antagonist; Wako, Tokyo, Japan), and ondansetron (5-HT_3_ receptor antagonist; Abcam, Tokyo, Japan), were dissolved in 10 µL of 100% dimethyl sulfoxide (DMSO; Sigma) [10,11].

### 2.3. Animal Model and Behavioral Test

**Figure 1:**
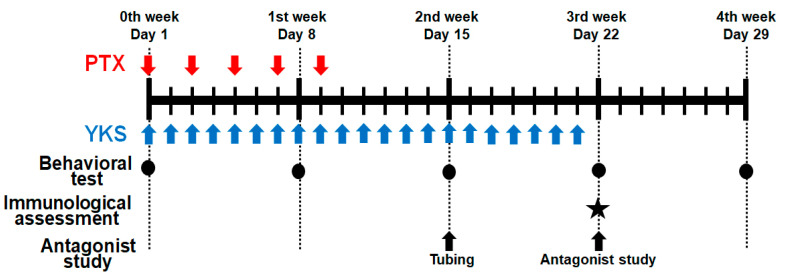
Schematic illustration of the experimental protocol. Male Sprague Dawley rats were treated with Paclitaxel (PTX) and/or Yokukansan (YKS). PTX was injected intraperitoneally with 2 mg/kg five times every 2 days. YKS was administered at 1 g/kg via an oral gastric tube daily for 3 weeks. Behavioral tests were performed on Day 1, before drug administration, Day 8, Day 15, Day 22, and Day 29. The immunological assessment was demonstrated on Day 22. Additionally, using PTX/YKS group rats, a study on 5-hydroxytryptamine receptor antagonists was performed on Day 22 after the tubing surgery on Day 15. PTX = Paclitaxel; YKS = Yokukansan. LL. ★: The immunological assessment was demonstrated on Day 22.

In the first experiment, the animals were randomized into the following four groups: the control group, PTX group, PTX/vehicle group, and PTX/YKS group (n = 10 in each group) (Figure 1).

Neuropathic pain was simulated in the PTX group with intraperitoneal injections of PTX (2 mg/kg) diluted with saline to 1 mL five times every 2 days on days 1, 3, 5, 7, and 9 (total 10 mg/kg) [12,13]. Rats in the control group were injected intraperitoneally with 1 mL of saline. PTX-induced neuropathic pain was assessed with the dynamic plantar aesthesiometer (Ugo Basile SRL, Gemonio, Italy) for automated mechanical stimulation and allodynia [12,14].

PTX rats were administered YKS (1 g/kg) or distilled water through an oral gastric tube every day for 3 weeks from the beginning of the experiment (PTX/YKS group or PTX/vehicle group) [15]. We compared paw withdrawal between the PTX/vehicle and PTX/YKS groups on Day 1, before YKS injection, Day 8 (1st week), Day 15 (2nd week), Day 22 (3rd week), and Day 29 (4th week).

Rats were acclimated in a restricted plastic cage with a dynamic plantar aesthesiometer on perforated wire platforms for a minimum of 15 min before starting the procedures. A movable touch-stimulator unit with a von Frey-type, 0.5 mm filament exerted increasing force vertically under the mid-plantar region of the hind paw until the animal withdrew its paw. We measured a paw withdrawal threshold six times, three times for each leg at intervals of a few minutes, and adopted four values of the paw withdrawal threshold, excluding the maximum and minimum values. The value was expressed in grams. A cut-off value of 50 g in 20 s was determined. Behavioral tests were performed on Day 1, before drug administration, Day 8 (1st week), Day 15 (2nd week), Day 22 (3rd week), and Day 29 (4th week).

### 2.4. Immunological Assessment of Glial Cell Changes via the Immunostaining of the Spinal Cord

Additionally, the immune response to changes in glial cells was evaluated in the PTX/vehicle group and the PTX/YKS group of rats on Day 22 (3rd week). Animals were anesthetized with an intravenous injection of thiamylal (20–30 mg) and 1% sevoflurane, and we performed a midline abdominal incision. Their diaphragms were perforated, and their pericardium was opened. A catheter was inserted in their left ventricular from their left apex. They were perfused using 300 mL of sterile 0.9% normal saline, and the right atrial appendage was cut simultaneously. After the effluent liquid became clear, 500 mL of 4% paraformaldehyde was used for perfusion until the tissues of the rats became hard. The L4/L5 segments of their spinal cords were exposed from the lumbar vertebral column via laminectomy. Samples of their lumbar enlargements were post-fixed in 4% paraformaldehyde [16,17].

To investigate glial reactivity, we used antibodies against ionized calcium-binding adaptor protein 1 (Iba1) to label microglia and the anti-Glial Fibrillary Acidic Protein (GFAP) to label astrocytes. The overexpression of Iba1 or GFAP is interpreted as a sign of microglial or astrocytic reactivity, as shown in different pain models [18,19].

The rats’ lumbar enlargements were washed twice in TBS. Primary antibodies were applied for 1 h at 4 °C with the following being the primary antibodies: the rabbit monoclonal antibody to GFAP was used to label astrocytes (1:500 dilution; Abcam (Waltham, MA, USA)); the goat polyclonal antibody to Iba1 was used to label microglia (1:200 dilution; Abcam, USA). Tissue sections were washed in TBS and then visualized with the appropriate secondary fluorescent antibody, chicken anti-rabbit IgG Alexa Fluor-488 or donkey anti-goat IgG Alexa Fluor-594.

The sections were examined using an electron microscope (ECLIPSE 90i, Nikon, Tokyo, Japan), and ImageJ (https://imagej.nih.gov/ij/ (accessed on 1 May 2019), National Institutes of Health, Bethesda, MD, USA) was used to quantify the GFAP-positive cells and the Iba-1-positive cells. The number of GFAP or Iba-1 positive cells from each animal was averaged to quantify GFAP or Iba-1-positive cells from five lumbar spinal cord section images. Five animals were allocated to each group. We examined the expression level of activated microglia between the PTX/vehicle group and the PTX/YKS group, counting the number of activated microglia with Iba1, a specific marker for microglia.

### 2.5. 5-HT Synthesis Inhibitors and 5-HT Receptor Antagonists

Next, using PTX/YKS group rats, we conducted experiments to clarify the involvement of serotonin. Four-Chloro-DL-phenylalanine methyl ester hydrochloride (PCPA), which depletes central serotonin (5-hydroxytryptamine, 5-HT), was injected intraperitoneally into the PTX/YKS group rats (PCPA group). PCPA (100 mg/kg) diluted with saline to 1 mL was administered for three days just before the behavior test [20,21]. Saline was injected as a vehicle in the control group. We compared the paw withdrawal threshold between the control group (n = 10) and the PCPA group (n = 5) with a dynamic plantar aesthesiometer.

On Day 22 (3rd week), we also conducted experiments to see how 5-HT receptor antagonists affected the PTX/YKS rats. We used WAY-100635 (5-HT_1A_ receptor antagonist), Ketanserin (5-HT_2A/2C_ receptor antagonist), and ondansetron (5-HT_3_ receptor antagonist) as the 5-HT receptor antagonists [10,11]. A polyethylene catheter (Intramedic PE-10, Becton Dickinson and CO., Franklin Lakes, NJ, USA) was implanted surgically in the PTX/YKS group rats under general anesthesia with an intravenous injection of thiamylal (20–30 mg/kg) on Day 15 (2nd week). The tip of the polyethylene catheter was positioned at the lumbar enlargement [10], and the opposite side of the catheter was withdrawn from the back of the neck via a subcutaneous tunnel. The catheter was attached to a stainless-steel wire, and the musculature and skin were sutured with a 3-0 silk suture thread. A one week recovery period was provided, and then the experiments resumed on Day 22 (3rd week).

PTX/YKS rats were injected intrathecally with 100% DMSO (vehicle) or 5-HT receptor antagonists: 60 µg of WAY-100635, 30 µg of Ketanserin, and 30 µg of ondansetron [10,11]. Each of the 5-HT receptor antagonists was dissolved in 10 µL of 100% DMSO. After the injection of each of the 5-HT receptor antagonists, 10 µL of saline was flushed. We evaluated the effect of the 5-HT receptor antagonists by measuring the paw withdrawal threshold with a dynamic plantar aesthesiometer 3 h after the injection of the 5-HT receptor antagonists.

### 2.6. Statistical Analysis

The experimental results are expressed as the mean and standard error of the mean (SEM). The statistical analysis was performed in GraphPad Prism 5.02 (GraphPad Software Inc., San Diego, CA, USA). The two groups were compared using the Mann–Whitney U test. A two-way analysis of variance (ANOVA) for multiple comparisons was used to analyze differences between groups. A *p*-value < 0.05 was considered to indicate statistical significance.

## 3. Results

### 3.1. Experimental Animals

Experiments were performed on 90 male Sprague Dawley rats. The animal model success rate of PTX rats was 95%. Animals that experienced nerve damage or deterioration in their overall condition were not included in the following experiment. Specifically, one animal from the PTX group, two from the PTX/YKS group, one from the PCPA group, and two from the antagonist study (from tubing to antagonist injection) were excluded.

### 3.2. Paclitaxel Effectively Decreases the Withdrawal Threshold

**Figure 2:**
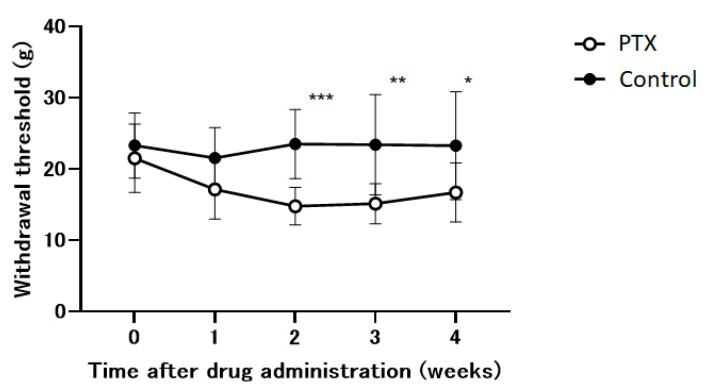
The time course of the withdrawal thresholds of the hind paw using a dynamic plantar aesthesiometer in Paclitaxel (PTX) or saline-administered (control) animals. Withdrawal thresholds were determined before administration and 1, 2, 3, and 4 weeks after the administration of the drug. Data are presented as mean ± SEM. * *p* < 0.05, ** *p* < 0.01, *** *p* < 0.001, compared with the respective baseline of the control group at the corresponding time points. n = 10 in each group.

Within our experimental framework, we utilize an animal model to study the neuropathic pain induced by PTX. PTX significantly reduced the withdrawal threshold during the initial two weeks and subsequently stabilizesd (Figure 2). This effect was not observed in the control group.

### 3.3. Yokukansan Improves Allodynia

**Figure 3:**
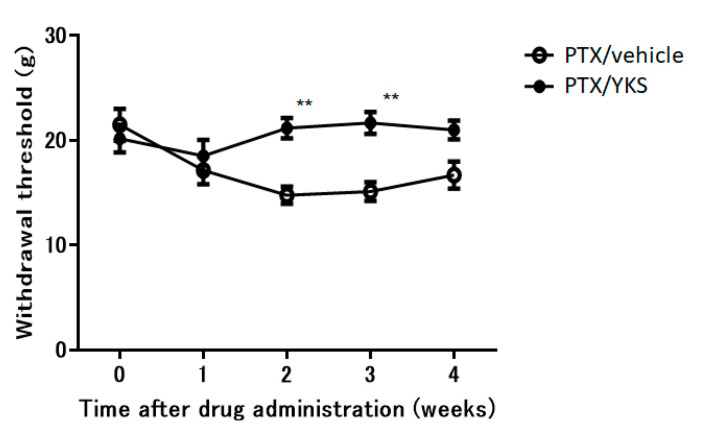
The time course of the effects of daily oral administration of water or Yokukansan on the withdrawal thresholds of the hind paw using a dynamic plantar aesthesiometer in Paclitaxel (PTX)-administered animals. Using PTX animals, withdrawal thresholds were determined before administration and 1, 2, 3, and 4 weeks after the administration of Yokukansan (PTX/YKS) or distilled water (PTX/vehicle). Data are presented as mean ± SEM. ** *p* < 0.01, compared with the respective baseline of the PTX/vehicle group at the corresponding time points. n = 10 in each group. PTX = Paclitaxel; YKS = Yokukansan.

There was no difference in weight gain between the PTX/YKS group and the PTX/vehicle group. YKS effectively improved allodynia by 30% (Figure 3). The PTX/YKS group had a significantly increased withdrawal threshold compared to the PTX/vehicle group during the second week [21.2 ± 1.0 g vs. 14.8 ± 0.8 g: *p* < 0.01] and the third week [21.7 ± 1.1 g vs. 15.1 ± 0.9 g: *p* < 0.01]. Interestingly, there was no significant difference between the PTX/YKS group and the PTX/vehicle group during the fourth week.

### 3.4. Yokukansan Prevents Microglia Growth

**Figure 4:**
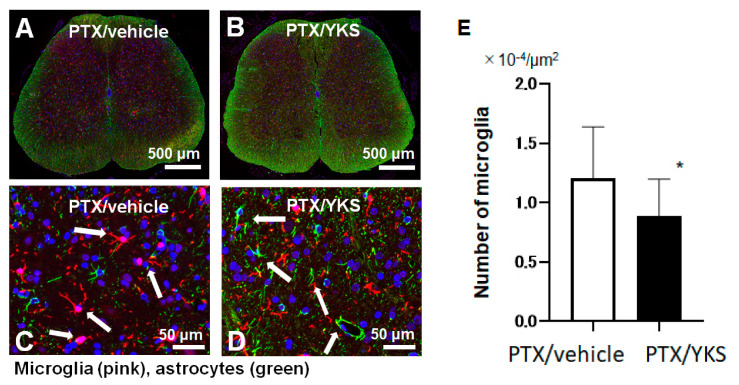
A comparison of the microglia expression levels between Paclitaxel/vehicle (PTX/vehicle) and Paclitaxel/Yokukansan (PTX/YKS). Fluorescent immunostaining of the spinal cord with Iba1, a specific marker of microglia (pink). Astrocytes were labeled with Anti-Glial Fibrillary Acidic Protein (green), and the nuclei were stained with DAPI (blue, 4′,6-diamidino-2-phenylindole). Panel (**A**) is a low-power view of the spinal cord of the PTX/vehicle group. Panel (**B**) is a low-power view of the PTX/YKS group. Panel (**C**,**D**) are high-power views of the PTX/vehicle and PTX/YKS groups, respectively. (**E**) The expression level of microglia was significantly suppressed. YKS = Yokukansan. * *p* < 0.05; n = 5 in each group.

In a comparison between the group of PTX rats that were administered YKS (PTX/YKS) and the PTX group that was administered distilled water (PTX/vehicle), the expression level of microglia was significantly suppressed by approximately 40% in the rats that were administered YKS ([0.8 × 10^4^ vs. 1.3 × 10^4^ µm^2^: *p* < 0.001], Figure 4).

### 3.5. Yokukansan Appears to Be Mediated by Serotonin in the Spinal Cord

**Figure 5:**
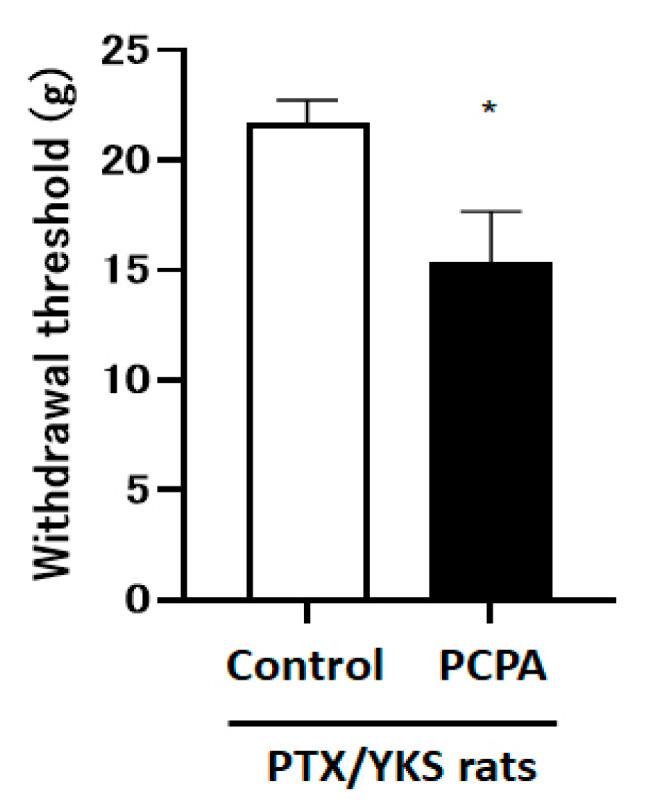
Serotonin depletion inhibits the effect of Yokukansan; this figure shows the effect of the intraperitoneal injection with 4-Chloro-DL-phenylalanine methyl ester hydrochloride (PCPA) on the anti-allodynic effect of Yokukansan. The animals were tested for sensory perception using a dynamic plantar aesthesiometer 3 weeks after being given Paclitaxel and Yokukansan (PTX/YKS). Animals received intraperitoneal injections of 300 mg/kg of PCPA (PCPA group) or a saline vehicle (control group). PCPA was administered daily for 3 days prior to the dynamic plantar test. Values are shown as the mean ± SEM. Control: n = 10, PCPA: n = 5. * *p* < 0.05, compared with the control group.

The intraperitoneal administration of 4-Chloro-DL-phenylalanine methyl ester hydrochloride (PCPA), which depletes central serotonin, decreased the paw withdrawal threshold compared with the control group [15.4 ± 2.3 vs. 21.7 ± 1.1 g: *p* < 0.05] (Figure 5).

### 3.6. Yokukansan Appears to Be Mediated via 5-HT_1A_ and 5-HT_2A/2C_ Receptors and Less Likely via 5-HT_3_

**Figure 6:**
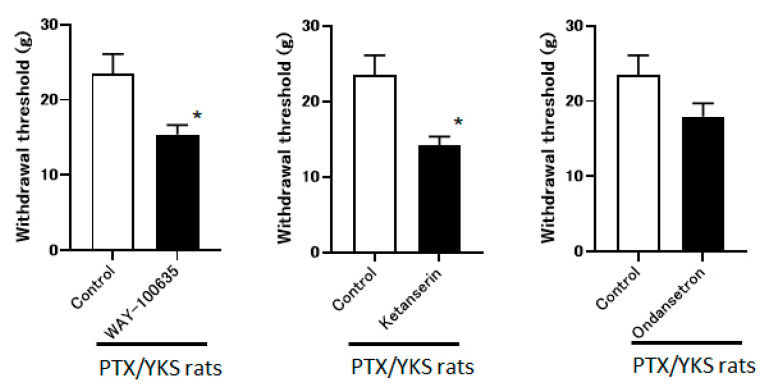
Yokukansan is mediated via 5-hydroxytryptamine (5-HT) receptors. This figure shows the effect of intrathecal injection with 5-HT receptor antagonists on the anti-allodynic effect of Yokukansan. The threshold was determined 3 weeks after Paclitaxel and Yokukansan (PTX/YKS) administration using a dynamic plantar aesthesiometer. The animals were administered an intrathecal injection of 60 μg of WAY100635 (a 5-HT_1A_ receptor antagonist), 30 μg of Ketanserin (a 5-TH_2A/2C_ receptor antagonist), 30 μg of ondansetron (a 5-HT3 receptor antagonist), or the control group received DMSO. The threshold was then assessed 60 min later. Values are shown as the mean ± SEM. Control, WAY-100635, and ondansetron: n = 6, Ketanserin: n = 7. * *p* < 0.05, compared with the control group.

The group that received the intrathecal administration of WAY-100635 and Ketanserin experienced a significant decrease in the withdrawal threshold (15.4 ± 1.2 g vs. 14.1 ± 1.2 g, respectively). The observed difference is statistically significant (*p* < 0.05) compared to the control group. However, the ondansetron did not observe these effects (17.9 ± 1.8 g). These data suggest that YKS appears to be mediated via the 5-HT_1A_ and 5-HT_2A/2C_ receptors and less likely via 5-HT_3_ (Figure 6).

## 4. Discussion

In this study, PTX effectively reduced the withdrawal threshold in our animal model of PTX-induced neuropathic pain (Figure 2). YKS improved allodynia by 30% and significantly increased the withdrawal threshold in PTX-administered animals during the second and third weeks (Figure 3). The efficacy of YKS may be mediated by serotonin in the spinal cord, especially through 5-HT_1A_ and 5-HT_2A/2C_ receptors, with less involvement of 5-HT_3_ receptors. However, in the fourth week, an increase in the paw withdrawal threshold of rats injected with PTX was observed, suggesting that neuropathic pain with a total of 10 mg/kg of PTX may be reversible (Figure 2).

Mechanical allodynia is characterized by pain perception responding to normally non-painful mechanical stimuli, such as a light touch or pressure. Animal models of mechanical allodynia are crucial for studying this condition’s underlying mechanisms and developing potential treatments. One common model used to study mechanical allodynia involves inducing nerve injury or inflammation in animals, such as rodents [12,17]. In this study, we used PTX to induce mechanical allodynia. The mechanism of the mechanical allodynia induced by PTX involves primarily neurotoxicity. PTX is a widely used chemotherapeutic agent for cancer treatment known to directly induce toxicity in nerve tissues. Several mechanisms of PTX-induced neurotoxicity have been reported. The main mechanisms are microtubule stabilization, apoptosis induction, and inflammation induction [22,23]. These mechanisms suggest that PTX-induced neurotoxicity contributes to the development of mechanical allodynia, a common side effect observed in patients during and after PTX treatment. Understanding its mechanism can aid in managing and treating this symptom.

YKS is a traditional Japanese medicine that can relieve neuropathic pain and other symptoms by regulating liver function and balancing energy in the body. PTX is a chemotherapy drug used to treat cancer, and its use is often associated with the development or worsening of neuropathic pain. There has not been sufficient research on how YKS affects the management of PTX-induced neuropathic pain. However, some preclinical studies suggest that YKS may modulate nervous system function and inflammatory responses, contributing to pain relief [24,25,26].

There is no fixed opinion regarding the administration method and dosage of YKS. Several experiments have demonstrated using YKS that was administered to rodents based on precise calculations of their food intake. The exact dose cannot be determined with that method, so we used a gastric tube to administer YKS. Regarding the dosage, Suzuki et al. reported the anti-allodynic effects on peripheral neuropathy using rats in a previous study, stating that the dosage of YKS was 0.3 g/kg and 1 g/kg [25]. This study’s 1 g/kg dosage was also based on previous similar studies [27,28]. Therefore, this dose of YKS is quite high in humans (it should be approximately 5–15 g/day for humans), but these animal experiments suggest that this dose is within an acceptable range.

The mechanism of neuropathic pain caused by Paclitaxel involves multiple factors, one of which is an inflammatory response in the nervous system. Paclitaxel causes inflammation within nerve tissue, which may play a role in the development of neuropathic pain [4,29]. Microglia play an important role in the inflammatory response of the nervous system and are thought to be activated when Paclitaxel triggers an inflammatory response in neural tissue. Additionally, astrocytes play an important role in the inflammatory response within neural tissue and may be activated when Paclitaxel causes inflammation in neural tissue [29]. Such inflammatory responses may contribute to the development and progression of neuropathic pain. Astrocytes are responsible for transmitting information to and from nerve cells and supplying nutrients, and the use of Paclitaxel may interfere with these functions, causing neuropathic pain [29]. Therefore, it is thought that astrocytes may play an important role in the relationship between neuropathic pain and Paclitaxel.

In animal models of schizophrenia, multiple sclerosis, and behavioral and psychological symptoms of dementia, YKS suppresses glial cell activity [30]. The activation of these glial cells is associated with the development and persistence of neuropathic pain, so glial cells and their associated molecules have become the targets of YKS treatment. Kawakami et al. reported that the administration of YKS inhibited the expression of activated astrocytes and astrogliosis [31]. Ebisawa et al. reported that YKS inhibited the increased expression of interleukin-6 in the dorsal horn of the spinal cord in the mouse model, and this expression was confirmed in astrocytes and/or microglia, not in neurons [24]. These studies suggest that YKS is effective against neuropathic pain, as evidenced by the regulation of microglial and astrocytic functions, which indicate the formula’s potential mechanisms.

This study revealed how YKS affects the peripheral neuropathy caused by PTX. The daily oral administration of YKS suppressed the expression level of activated microglia. Previous studies revealed that peripheral nerve injury leads to a series of microglia changes within the spinal cord’s dorsal horn, and microglia in the spinal cord convert from a resting state to an activated state and start proliferation [19,32]. Therefore, this study showed that the suppression of the expression level of activated microglia indicates that YKS was effective against Paclitaxel-induced neuropathic pain.

The mechanism of serotonin’s analgesic effect involves the modulation of pain signaling pathways in the central nervous system. Serotonin, acting through its various receptor subtypes, can inhibit the transmission of pain signals by suppressing the activity of pain-sensing neurons, enhancing the release of endogenous opioids, and modulating the activity of descending pain inhibition pathways [9]. Additionally, serotonin can exert anti-inflammatory effects, contributing to its analgesic properties [33]. The dysfunction of the descending pain modulatory system reportedly involves the development of chronic pain. YKS acts as an agonist of the 5-HT_1A_ receptor; geissoschizine methyl ether, an alkaloid synthesized by the YKS component Uncaria hook, is believed to play this role [27,34].

Recent studies of YKS for neuropathic pain confirm that YKS relieves mechanical allodynia in partial sciatic nerve ligation mice by regulating the expression of interleukin-6 in astrocytes and/or microglia in the spinal cord [24] and that YKS has anti-allodynic effects in rats with chronic contraction injuries, which are associated with the blockade of glutamatergic neurotransmission via the activation of glutamate transporters in the spinal cord [25]. Nakao et al. have shown that YKS alleviates cancer pain via suppressing matrix metalloproteinase-9 expression in the bone metastasis model in mice [35]. The present data show for the first time that YKS affects PTX-induced peripheral neuropathy instead of physical peripheral neuropathy. PTX is one of the most widely used anticancer drugs, and it has been reported that many patients receiving PTX may experience peripheral neuropathy as a side effect [4]. Therefore, it would be very important to demonstrate the effect of YKS on PTX-induced peripheral neuropathy.

Additionally, in this study, the anti-allodynic effect of YKS was inhibited following serotonin depletion via the intraperitoneal administration of a serotonin synthesis inhibitor. This highly suggests that serotonin is involved in the effects of YKS on PTX-induced peripheral neuropathy. YKS has also been reported to affect the 5-HT nervous system and has been shown to act as a partial agonist on the 5-HT_1A_ receptor [9] and to induce the downregulation of the 5-HT_2A_ receptor in the prefrontal cortex, which suggests the involvement of the 5-HT system in the psychopharmacological effects of YKS [36]. Most previous reports on the 5-HT effects of YKS have focused on its anxiolytic effects [37,38], reduction of emotional abnormalities [39], and amelioration of aggressiveness and sociality [27,40], but our results showed for the first time that 5-HT is involved in the effects of YKS on PTX-induced peripheral neuropathy. In particular, the intrathecal administration of WAY-100635 and Ketanserin decreased the withdrawal threshold, suggesting that 5-HT_1A_ receptors and 5-HT_2A/2C_ receptors in the spinal cord act on the anti-allodynic effect of YKS. Although it was unclear whether YKS ameliorated the dysfunction of the descending pain regulatory system, this study suggests that YKS regulates the excitability of the 5-HT nervous system and suppresses descending pain in the spinal cord.

There are some limitations to this study. YKS suppresses mechanical allodynia in the neuropathic pain model, and its effect is reported to involve the activation of glutamate transporters, which functionally uptake glutamate [25]. Further investigation is needed to identify if the anti-allodynic effect of YKS on PTX-induced neuropathic pain may involve the activation of glutamate transporters. Additionally, transient receptor potential (TRP) vanilloid 1, to which capsaicin binds, and TRP vanilloid 8 receptors, to which cold stimuli are received, are involved in the neuropathic pain caused by PTX [41,42]. Whether YKS has TRP channel activity requires future investigation, as other Japanese herbal medicines like Kampo have been shown to have TRP activity. This study conducted experiments during the third week when the anti-allodynic effect of YKS was most pronounced. PTX and YKS were started at the same time, and it is unclear whether YKS treated or prevented Paclitaxel-induced neuropathic pain. Further investigation is necessary to identify the impact of the timing of the administration of YKS. Furthermore, YKS contains seven herbs, each with its ingredients, and their effects have been studied. In this study, we used YKS but did not mention the actions of each ingredient.

## 5. Conclusions

Our results suggest that the oral administration of Yokukansan resulted in an anti-allodynic effect in an animal model of Paclitaxel-induced neuropathic pain. Furthermore, Yokukansan may play an important role in alleviating the peripheral neuropathy associated with cancer chemotherapy via a 5-HT-mediated mechanism.

## Figures and Tables

**Table 1:** The component herbs of Yokukansan.

Atractylodes lancea rhizome	4.0 g
Poria sclerotium	4.0 g
Cnidium rhizome	3.0 g
Uncaria hook	3.0 g
Angelica root	3.0 g
Bupleurum root	2.0 g
Glycyrrhiza	1.5 g

The weights indicate the relative amounts mixed. Contains magnesium, stearate, and lactose hydrate as additives.

## Data Availability

The data to support the findings of this study are available from the corresponding author upon request.

## References

[1] Staff N.P., Grisold A., Grisold W., Windebank A.J. (2017). Chemotherapy-induced peripheral neuropathy: A current review. Ann. Neurol..

[2] Weaver B.A. (2014). How Taxol/paclitaxel kills cancer cells. Mol. Biol. Cell.

[3] Sharifi-Rad J., Quispe C., Patra J.K., Singh Y.D., Panda M.K., Das G., Adetunji C.O., Michael O.S., Sytar O., Polito L. (2021). Paclitaxel: Application in Modern Oncology and Nanomedicine-Based Cancer Therapy. Oxid. Med. Cell Longev..

[4] Staff N.P., Fehrenbacher J.C., Caillaud M., Damaj M.I., Segal R.A., Rieger S. (2020). Pathogenesis of paclitaxel-induced peripheral neuropathy: A current review of in vitro and in vivo findings using rodent and human model systems. Exp. Neurol..

[5] Bocci G., Di Paolo A., Danesi R. (2013). The pharmacological bases of the antiangiogenic activity of paclitaxel. Angiogenesis..

[6] Sunagawa M., Takayama Y., Kato M., Tanaka M., Fukuoka S., Okumo T., Tsukada M., Yamaguchi K. (2021). Kampo Formulae for the Treatment of Neuropathic Pain approximately Especially the Mechanism of Action of Yokukansan approximately. Front. Mol. Neurosci..

[7] Mizoguchi K., Ikarashi Y. (2017). Multiple Psychopharmacological Effects of the Traditional Japanese Kampo Medicine Yokukansan, and the Brain Regions it Affects. Front. Pharmacol..

[8] Kawada K., Ishida T., Jobu K., Morisawa S., Kawazoe T., Nishida M., Nishimura S., Tamura N., Yoshioka S., Miyamura M. (2022). Yokukansan suppresses neuroinflammation in the hippocampus of mice and decreases the duration of lipopolysaccharide- and diazepam-mediated loss of righting reflex induced by pentobarbital. J. Nat. Med..

[9] Terawaki K., Ikarashi Y., Sekiguchi K., Nakai Y., Kase Y. (2010). Partial agonistic effect of yokukansan on human recombinant serotonin 1A receptors expressed in the membranes of Chinese hamster ovary cells. J. Ethnopharmacol..

[10] Kato T., Kajiyama S., Hamada H., Kawamoto M. (2013). Long-term administration of fluvoxamine attenuates neuropathic pain and involvement of spinal serotonin receptors in diabetic model rats. Hiroshima J. Med. Sci..

[11] Obata H., Saito S., Sasaki M., Ishizaki K., Goto F. (2001). Antiallodynic effect of intrathecally administered 5-HT(2) agonists in rats with nerve ligation. Pain.

[12] Thangamani D., Edafiogho I.O., Masocha W. (2013). The anticonvulsant enaminone E139 attenuates paclitaxel-induced neuropathic pain in rodents. Sci. World J..

[13] Chen N., Ge M.M., Li D.Y., Wang X.M., Liu D.Q., Ye D.W., Tian Y.K., Zhou Y.Q., Chen J.P. (2021). beta2-adrenoreceptor agonist ameliorates mechanical allodynia in paclitaxel-induced neuropathic pain via induction of mitochondrial biogenesis. Biomed. Pharmacother..

[14] Al-Romaiyan A., Masocha W. (2022). Pristimerin, a triterpene that inhibits monoacylglycerol lipase activity, prevents the development of paclitaxel-induced allodynia in mice. Front. Pharmacol..

[15] Furuya M., Miyaoka T., Tsumori T., Liaury K., Hashioka S., Wake R., Tsuchie K., Fukushima M., Ezoe S., Horiguchi J. (2013). Yokukansan promotes hippocampal neurogenesis associated with the suppression of activated microglia in Gunn rat. J. Neuroinflamm..

[16] Yang H., Wu L., Deng H., Chen Y., Zhou H., Liu M., Wang S., Zheng L., Zhu L., Lv X. (2020). Anti-inflammatory protein TSG-6 secreted by bone marrow mesenchymal stem cells attenuates neuropathic pain by inhibiting the TLR2/MyD88/NF-kappaB signaling pathway in spinal microglia. J. Neuroinflamm..

[17] Zeng H., Liu N., Yang Y.Y., Xing H.Y., Liu X.X., Li F., La G.Y., Huang M.J., Zhou M.W. (2019). Lentivirus-mediated downregulation of alpha-synuclein reduces neuroinflammation and promotes functional recovery in rats with spinal cord injury. J. Neuroinflamm..

[18] Romero-Sandoval A., Eisenach J.C. (2007). Spinal cannabinoid receptor type 2 activation reduces hypersensitivity and spinal cord glial activation after paw incision. Anesthesiology.

[19] Pottorf T.S., Rotterman T.M., McCallum W.M., Haley-Johnson Z.A., Alvarez F.J. (2022). The Role of Microglia in Neuroinflammation of the Spinal Cord after Peripheral Nerve Injury. Cells.

[20] Schechter M.D. (1991). Effect of serotonin depletion by p-chlorophenylalanine upon discriminative behaviours. Gen. Pharmacol..

[21] Sinha R.K. (2006). P-CPA pretreatment reverses the changes in sleep and behavior following acute immobilization stress rats. J. Physiol. Sci..

[22] Sanchez-Carranza J.N., Redondo-Horcajo M., Barasoain I., Escobar-Aguilar E.A., Millan-Pacheco C., Alvarez L., Salas Vidal E., Diaz J.F., Gonzalez-Maya L. (2023). Tannic Acid and Ethyl Gallate Potentialize Paclitaxel Effect on Microtubule Dynamics in Hep3B Cells. Pharmaceuticals.

[23] Yardim A., Kandemir F.M., Comakli S., Ozdemir S., Caglayan C., Kucukler S., Celik H. (2021). Protective Effects of Curcumin Against Paclitaxel-Induced Spinal Cord and Sciatic Nerve Injuries in Rats. Neurochem. Res..

[24] Ebisawa S., Andoh T., Shimada Y., Kuraishi Y. (2015). Yokukansan Improves Mechanical Allodynia through the Regulation of Interleukin-6 Expression in the Spinal Cord in Mice with Neuropathic Pain. Evid. Based Complement. Alternat. Med..

[25] Suzuki Y., Mitsuhata H., Yuzurihara M., Kase Y. (2012). Antiallodynic effect of herbal medicine yokukansan on peripheral neuropathy in rats with chronic constriction injury. Evid. Based Complement. Alternat. Med..

[26] Yamaguchi K., Yamazaki S., Kumakura S., Someya A., Iseki M., Inada E., Nagaoka I. (2020). Yokukansan, a Japanese Herbal Medicine, Suppresses Substance PInduced Production of Interleukin-6 and Interleukin-8 by Human U373 MG Glioblastoma Astrocytoma Cells. Endocr. Metab. Immune Disord. Drug Targets.

[27] Nishi A., Yamaguchi T., Sekiguchi K., Imamura S., Tabuchi M., Kanno H., Nakai Y., Hashimoto K., Ikarashi Y., Kase Y. (2012). Geissoschizine methyl ether, an alkaloid in Uncaria hook, is a potent serotonin (1)A receptor agonist and candidate for amelioration of aggressiveness and sociality by yokukansan. Neuroscience.

[28] Imamura S., Tabuchi M., Oizumi H., Ueki T., Omiya Y., Ikarashi Y., Mizoguchi K. (2020). Yokukansankachimpihange, a traditional Japanese (Kampo) medicine, enhances the adaptation to circadian rhythm disruption by increasing endogenous melatonin levels. J. Pharmacol. Sci..

[29] Xu Y., Jiang Z., Chen X. (2022). Mechanisms underlying paclitaxel-induced neuropathic pain: Channels, inflammation and immune regulations. Eur. J. Pharmacol..

[30] Mizoguchi K., Ikarashi Y. (2017). Cellular Pharmacological Effects of the Traditional Japanese Kampo Medicine Yokukansan on Brain Cells. Front. Pharmacol..

[31] Kawakami Z., Ikarashi Y., Kase Y. (2010). Glycyrrhizin and its metabolite 18 beta-glycyrrhetinic acid in glycyrrhiza, a constituent herb of yokukansan, ameliorate thiamine deficiency-induced dysfunction of glutamate transport in cultured rat cortical astrocytes. Eur. J. Pharmacol..

[32] Tsuda M., Inoue K., Salter M.W. (2005). Neuropathic pain and spinal microglia: A big problem from molecules in “small” glia. Trends Neurosci..

[33] Zhao Y.L., Xu J.L., Yi H.Y., Baba S.S., Guo Y.X., Hou X.M., Yuan X.C., Li X.H., Wang Y.Y., Liang L.L. (2024). Activation of 5-HT(5A) receptor in the ventrolateral orbital cortex produces antinociceptive effects in rat models of neuropathic and inflammatory pain. Neuropharmacology.

[34] Ikarashi Y., Sekiguchi K., Mizoguchi K. (2018). Serotonin Receptor Binding Characteristics of Geissoschizine Methyl Ether, an Indole Alkaloid in Uncaria Hook. Curr. Med. Chem..

[35] Nakao K., Fujiwara A., Komasawa N., Jin D., Kitano M., Matsunami S., Takai S., Ito S., Minami T. (2019). Yokukansan Alleviates Cancer Pain by Suppressing Matrix Metalloproteinase-9 in a Mouse Bone Metastasis Model. Evid. Based Complement. Alternat. Med..

[36] Egashira N., Iwasaki K., Ishibashi A., Hayakawa K., Okuno R., Abe M., Uchida N., Mishima K., Takasaki K., Nishimura R. (2008). Repeated administration of Yokukansan inhibits DOI-induced head-twitch response and decreases expression of 5-hydroxytryptamine (5-HT)2A receptors in the prefrontal cortex. Prog. Neuropsychopharmacol. Biol. Psychiatry.

[37] Yamaguchi T., Tsujimatsu A., Kumamoto H., Izumi T., Ohmura Y., Yoshida T., Yoshioka M. (2012). Anxiolytic effects of yokukansan, a traditional Japanese medicine, via serotonin 5-HT1A receptors on anxiety-related behaviors in rats experienced aversive stress. J. Ethnopharmacol..

[38] Ohno R., Miyagishi H., Tsuji M., Saito A., Miyagawa K., Kurokawa K., Takeda H. (2018). Yokukansan, a traditional Japanese herbal medicine, enhances the anxiolytic effect of fluvoxamine and reduces cortical 5-HT(2A) receptor expression in mice. J. Ethnopharmacol..

[39] Tsuji M., Takeuchi T., Miyagawa K., Ishii D., Imai T., Takeda K., Kitajima M., Takeda H. (2014). Yokukansan, a traditional Japanese herbal medicine, alleviates the emotional abnormality induced by maladaptation to stress in mice. Phytomedicine.

[40] Ueki T., Mizoguchi K., Yamaguchi T., Nishi A., Ikarashi Y., Hattori T., Kase Y. (2015). Yokukansan Increases 5-HT1A Receptors in the Prefrontal Cortex and Enhances 5-HT1A Receptor Agonist-Induced Behavioral Responses in Socially Isolated Mice. Evid. Based Complement. Alternat. Med..

[41] Pittman S.K., Gracias N.G., Vasko M.R., Fehrenbacher J.C. (2014). Paclitaxel alters the evoked release of calcitonin gene-related peptide from rat sensory neurons in culture. Exp. Neurol..

[42] Materazzi S., Fusi C., Benemei S., Pedretti P., Patacchini R., Nilius B., Prenen J., Creminon C., Geppetti P., Nassini R. (2012). TRPA1 and TRPV4 mediate paclitaxel-induced peripheral neuropathy in mice via a glutathione-sensitive mechanism. Pflugers Arch..

